# miR-1290 promotes lung adenocarcinoma cell proliferation and invasion by targeting SOCS4

**DOI:** 10.18632/oncotarget.24046

**Published:** 2018-01-08

**Authors:** Xuelian Xiao, Daheng Yang, Xue Gong, Dongping Mo, Shiyang Pan, Jian Xu

**Affiliations:** ^1^ Department of Laboratory Medicine, The First Affiliated Hospital of Nanjing Medical University, Nanjing 210029, China; ^2^ National Key Clinical Department of Laboratory Medicine, Nanjing 210029, China

**Keywords:** lung adenocarcinoma, miR-1290, SOCS4, proliferation, invasion

## Abstract

miRNAs play important roles in lung adenocarcioma (LADC) progression. We previously found that miR-1290 expression was upregulated in LADC tissue and serum samples from patients with LADC, and correlated with prognosis. However, the biological role of miR-1290 in LADC and mechanism of such role are poorly understood. Here, we found that miR-1290 overexpression promoted LADC cell proliferation, cell cycle progression and invasion, while suppressing cell apoptosis *in vitro*. Furthermore, miR-1290 promoted tumor growth, invasion and metastasis *in vivo*. miR-1290 downregulated suppressor of cytokine signaling 4 (SOCS4) at both the mRNA and protein levels by targeting SOCS4. Reduced SOCS4 level reversed the inhibitory effect of miR-1290 downregulation on cell proliferation and invasion. miR-1290 activated the JAK/STAT3 and PI3K/AKT signaling pathways by targeting SOCS4. An inverse correlation was observed between miR-1290 and SOCS4 expression in LADC tissues. Clinicopathological characteristics analysis showed that SOCS4 expression was negatively associated with higher clinical stage and lymph node metastasis. These observations suggest that miR-1290 promotes LADC cell proliferation and invasion by targeting SOCS4.

## INTRODUCTION

Lung cancer is the leading cause of cancer mortality worldwide, and lung adenocarcinoma (LADC) accounts for more than 40%–45% of lung cancer cases [1, 2]. Despite advances in detection and standard treatments, LADC is often diagnosed at an advanced stage and the prognosis is not optimistic, with a five-year survival rate of approximately 15% [2, 3]. Rapid tumor proliferation and lethal distant metastasis are mainly responsible for failure of therapy and poor prognosis. A better understanding of the molecular mechanisms underlying tumor origins and progression would contribute to cancer diagnosis and therapy.

MicroRNAs (miRNAs) are small, single-stranded, non-coding RNAs consisting of 20–24 nucleotides, which lead to translational repression or mRNA degradation by binding to the 3′-untranslated regions (3′-UTRs) of target mRNAs [4, 5]. Increasing evidence has emerged that miRNAs participate in various biological processes in tumors, including cell proliferation [6], apoptosis [7], invasion and metastasis [8]. miRNAs can function as either tumor suppressors or oncogenes in cancers such as lung cancer [6], hepatocellular carcinoma [9] and ovarian cancer [10]. Elucidation of the role of miRNAs in cancer can give new perspectives to cancer diagnosis and therapy [11, 12].

Expression of many miRNAs is disrupted in LADC [13], and accumulating evidence has validated a potential role of miRNA in LADC diagnosis and therapy [14]. We previously showed that miR-1290 expression was higher in LADC tissues and serum samples than the controls, and that miR-1290 may be a potential prognostic biomarker for LADC [15]. However, the effects of miR-1290 on LADC and the mechanisms of such effects are poorly understood.

## RESULTS

### miR-1290 promotes lung adenocarcinoma cell proliferation and cell cycle progression, and suppresses cell apoptosis

**Figure 1:**
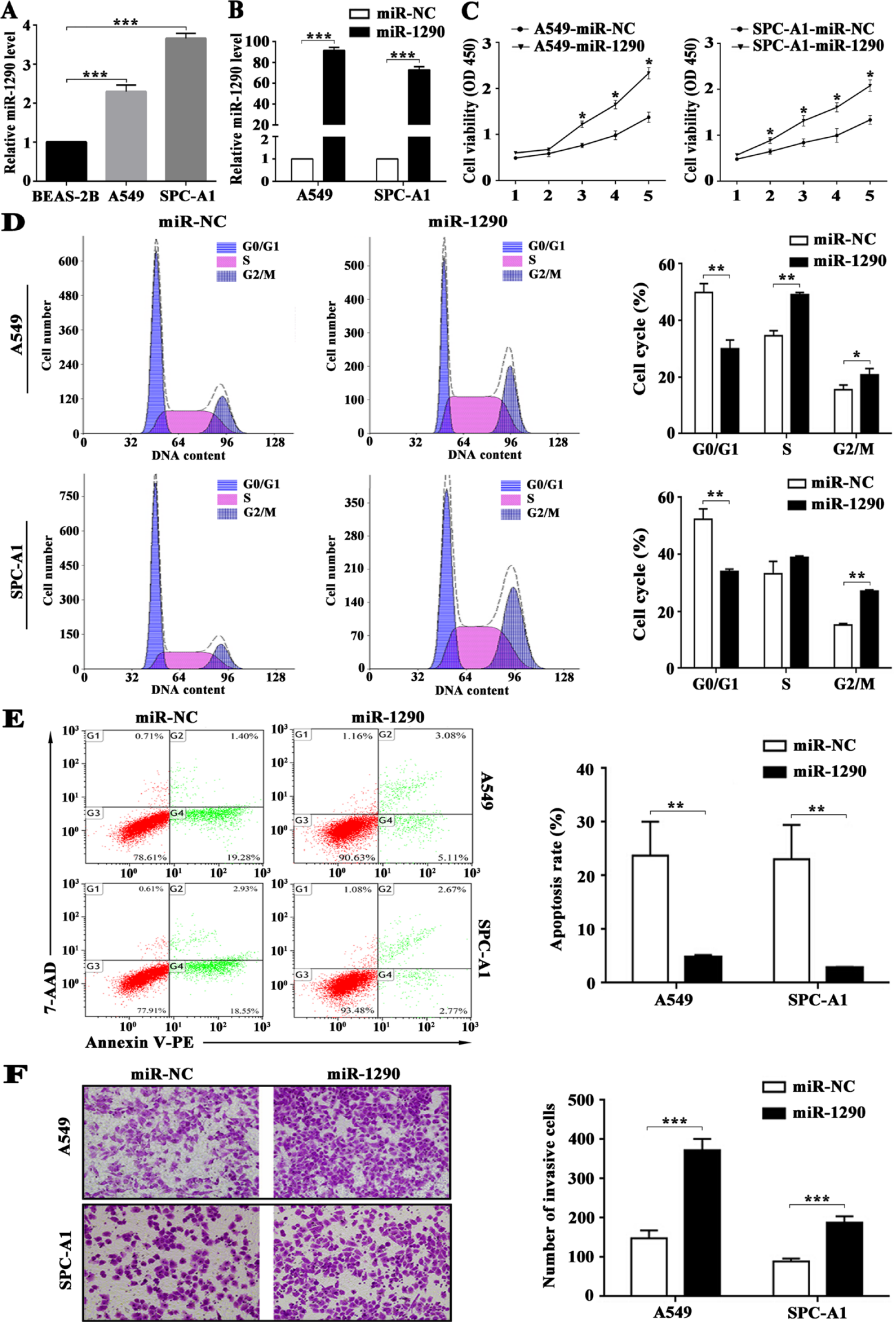
miR-1290 promotes proliferation, cell cycle progression and invasion, and suppresses apoptosis of lung adenocarcinoma cells A549 and SPC-A1 cells were transiently transfected with miR-1290 mimic or miR-NC. (**A**) miR-1290 expression levels in BEAS-2B, A549 and SPC-A1 cells were detected by qRT-PCR. (**B**) miR-1290 expression levels in the cells were detected by qRT-PCR. (**C**) Cell viability was determined by CCK-8 assy. (**D**) Cell cycle distribution was measured by propidium iodide staining and flow cytometry. (**E**) Cell Apoptosis was determined by Annexin V/PE and 7-AAD staining and flow cytometry; the apoptosis rate is presented on the right. (**F**) Cell invasiveness was detected by transwell invasion assay; representative images are shown on the left, and invasive cell number based on 10 randomly selected fields is shown on the right. Data is presented as mean ± standard deviation (SD). ^*^P < 0.05, ^**^P < 0.01, ^***^P < 0.001.

We previously demonstrated significantly increased expression of miR-1290 in both LADC tissues and serum samples compared with corresponding controls [15]. These findings inspired us to investigate the biological functions of miR-1290 in LADC. qRT-PCR analysis showed that miR-1290 levels were significantly higher in A549 and SPC-A1 cells than that in BEAS-2B cells (Figure 1A). Furthermore, qRT-PCR analysis revealed that miR-1290 levels were significantly higher in A549 and SPC-A1 cells transfected with miR-1290 mimic than that in controls (Figure 1B). Cell Counting Kit-8 (CCK-8) assay demonstrated that miR-1290 overexpression significantly promoted cell proliferation (Figure 1C). Furthermore, cell cycle analysis showed that miR-1290 overexpression significantly increased the percentage of cells in the G2/M phase while decreasing the percentage of cells in the G1/G0 peak (Figure 1D), which indicated that miR-1290 promoted cell cycle progression. Additionally, cell apoptosis decreased in cells transfected with miR-1290 mimic compared with controls (Figure 1E). Taken together, these findings implied that miR-1290 acted as a potential pro-tumor factor in the progression of LADC.

### miR-1290 promotes lung adenocarcinoma cell invasion *in vitro*

We previously found that miR-1290 expression was positively correlated with tumor stage and lymph node metastasis [15]. Here, we performed transwell assay to explore the effects of miR-1290 on cell invasion. We observed that miR-1290 overexpression markedly enhanced the invasiveness of A549 and SPC-A1 cells (Figure 1F).

### miR-1290 promotes tumor growth, invasion and metastasis *in vivo*

**Figure 2:**
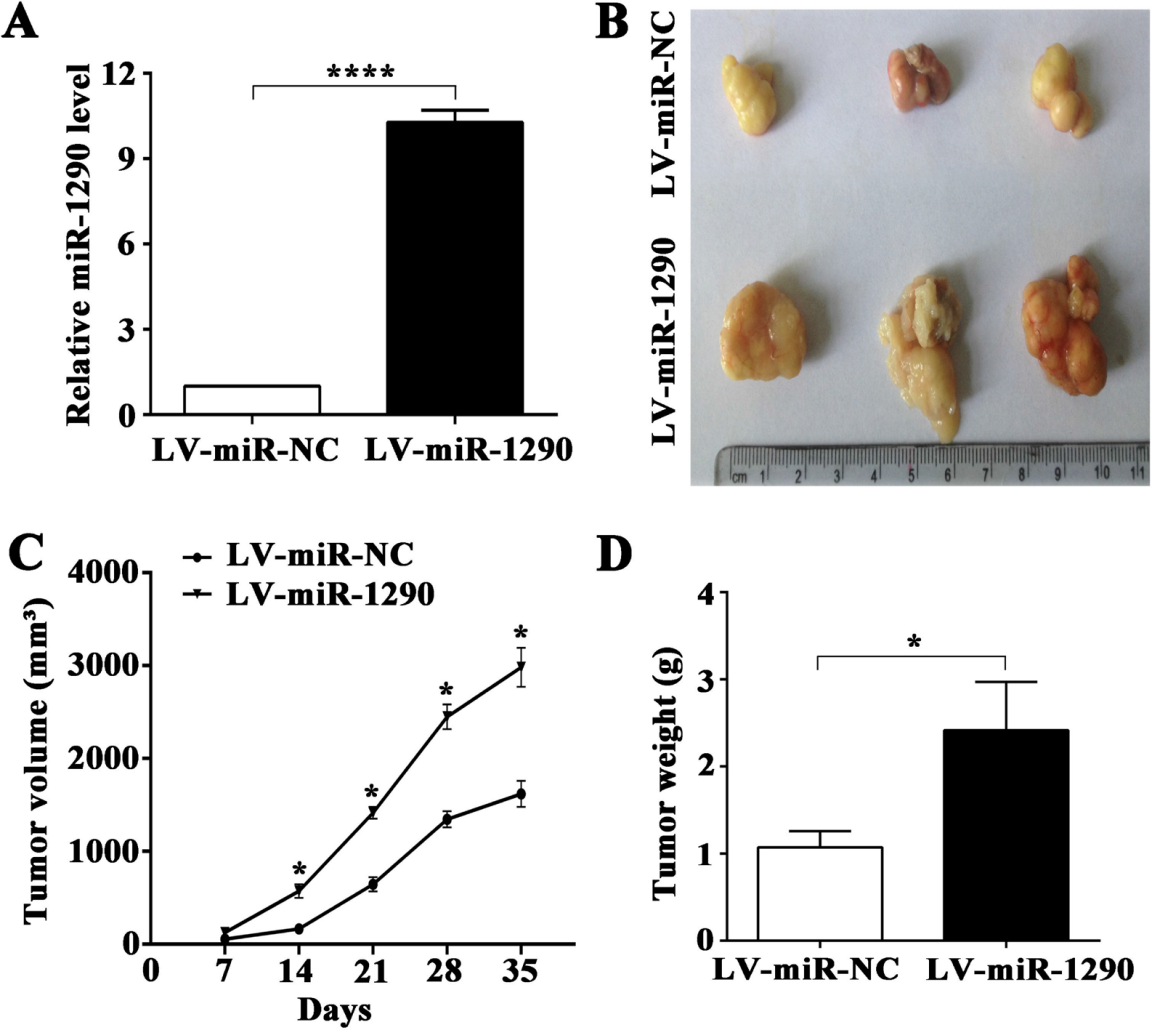
miR-1290 promotes tumor growth *in vivo* (**A**) miR-1290 expression in SPC-A1 cells infected with a lentiviral vector (LV) bearing miR-1290 (LV-miR-1290) or miR-NC (LV-miR-NC) was detected by qRT-PCR. Tumor size (**B**) and weight (**D**) were determined at day 35 in nude mice subcutaneously injected with LV-miR-1290 or LV-miR-NC-infected cells. Tumor growth curves (**C**) were shown as indicated. Data is presented as mean ± SD of 3 mice. ^*^P < 0.05, ^****^P < 0.0001.

In an effort to investigate the pro-tumor role of miR-1290 *in vivo*, luciferase-labeled SPC-A1 cells transfected with lentiviral vector (LV) bearing miR-1290 (LV-miR-1290) or miRNA control (LV-miR-NC) were subcutaneously injected into the right flank of nude mice (*n =* 3). miR-1290 expression was significantly higher in LV-miR-1290-infected cells than that in controls (Figure 2A). Tumor growth was subsequently monitored for 35 days. After 35 days, tumor volume and weight were both significantly higher in LV-miR-1290 group than in LV-miR-NC group (tumor volume, 1618 ± 80.62 mm^3^ vs 2982 ± 121.60 mm^3^, *P <* 0.001, Figure 2B; and weight, 1.072 ± 0.107 g vs 2.415 ± 0.321 g, *P <* 0.05, Figure 2D). Throughout the tumorigenesis process, tumors in the nude mice injected with LV-miR-1290-infected cells grew more rapidly than those in mice injected with LV-miR-NC-infected cells (Figure 2C). These results demonstrated that miR-1290 promoted tumor growth *in vivo*.

**Figure 3:**
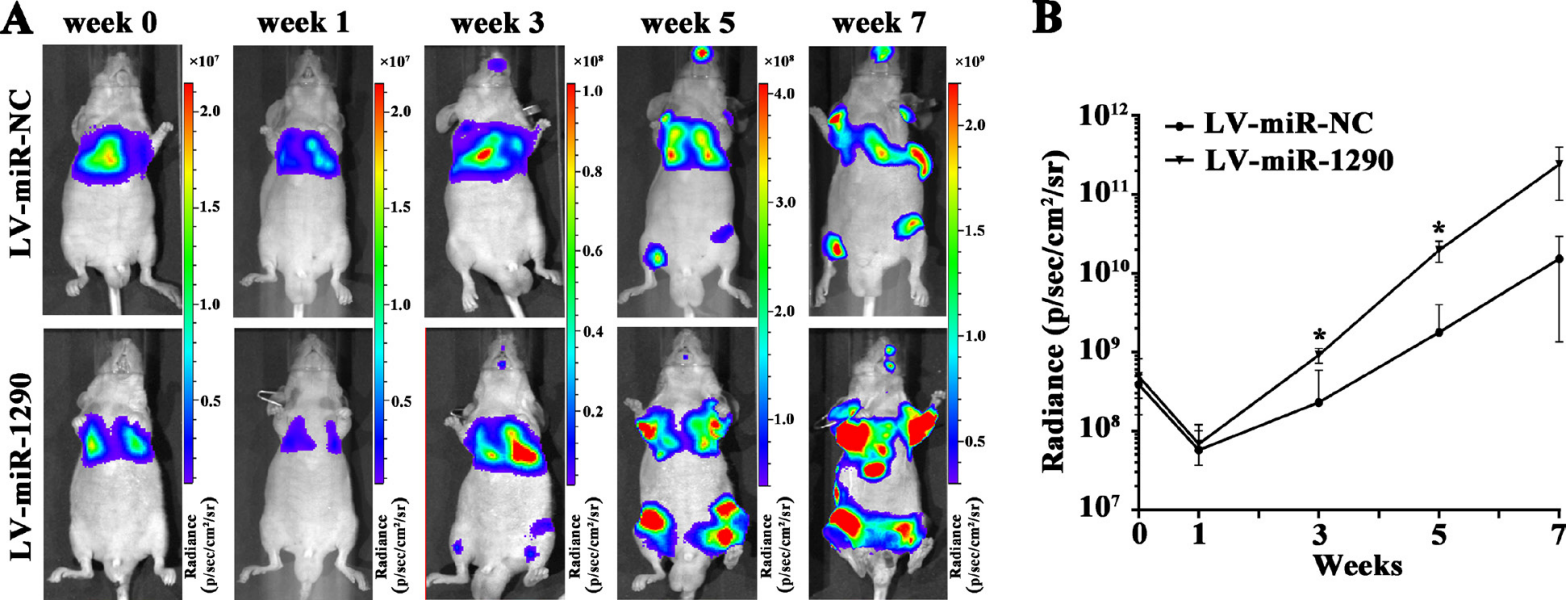
miR-1290 promotes tumor growth, invasion and metastasis *in vivo* Luciferase-labeled SPC-A1 cells transfected with LV-miR-1290 or LV-miR-NC were injected into the tail veins of nude mice. (**A**) Representative bioluminescence images of tumors in nude mice at week 0, 1, 3, 5 and 7. (**B**) Luminescence was assessed in 3 mice of each group at week 0, 1, 3, 5 and 7 after injection. Data is presented as mean ± SD. ^*^P < 0.05.

We next assessed the effects of miR-1290 on tumor invasion and metastasis *in vivo*. Luciferase-labeled SPC-A1 cells infected with LV-miR-1290 or LV-miR-NC were injected into the tail veins of nude mice (*n =* 3). Bioluminescence imaging was performed to monitor tumor aggressiveness for 7 weeks. Approximately equal levels of bioluminescence signal were observed in miR-NC and miR-1290 groups initially, followed by decreased luminescence at week 1, probably because of physiological metabolism. Subsequently, the luminescence increased as tumor progressed. Compared with the LV-miR-NC group, nude mice injected with LV-miR-1290-infected cells exhibited more intense and disseminated bioluminescence signal at week 3, 5 and 7, which suggested that miR-1290 promoted tumor growth, invasion and metastasis (Figure 3).

### miR-1290 directly targets suppressor of cytokine signaling 4 (SOCS4)

**Figure 4:**
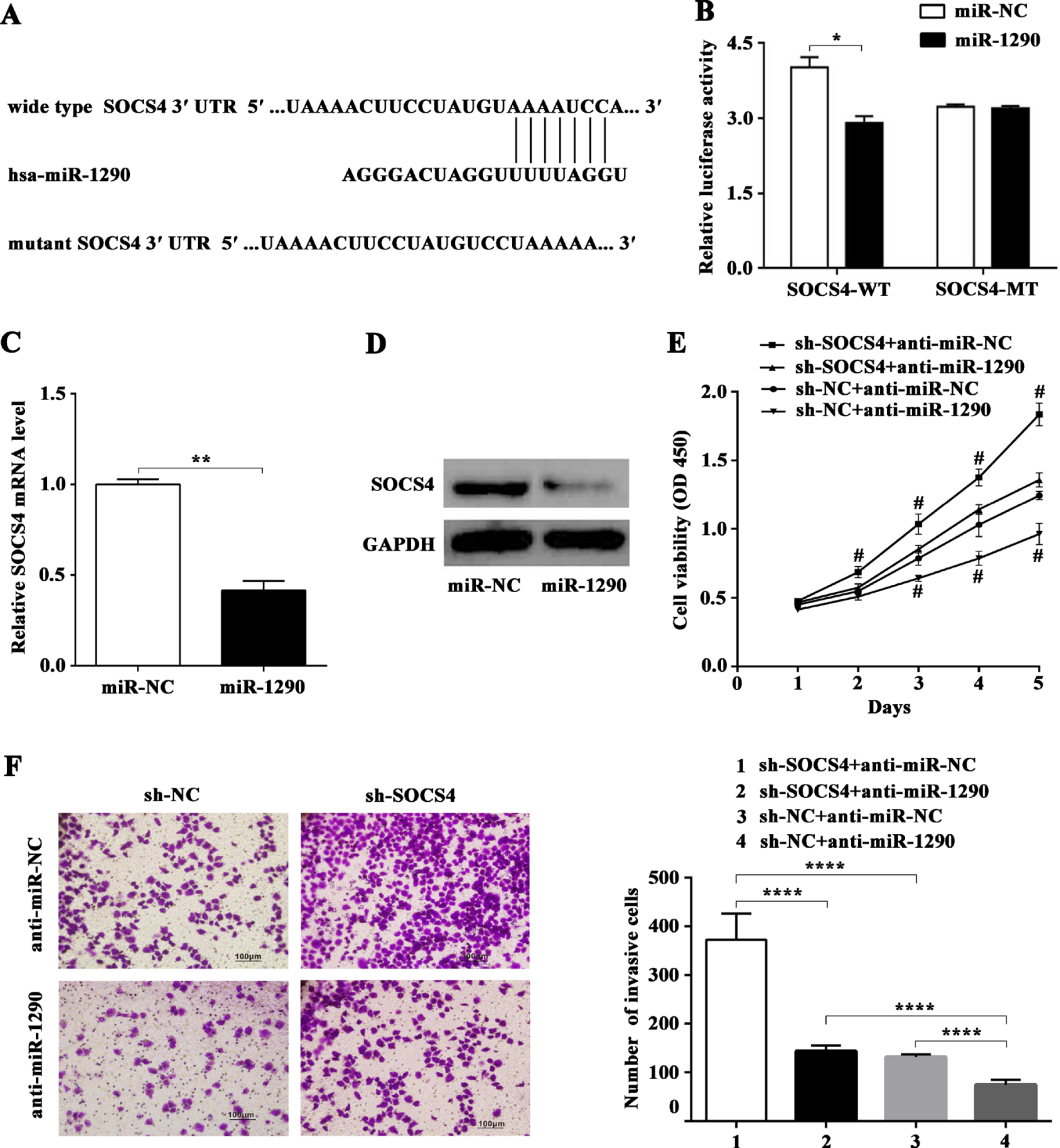
miR-1290 promotes cell proliferation and invasion in lung adenocarcinoma by targeting SOCS4 (**A**) The putative binding sites for miR-1290 in the 3′-UTR of SOCS4 were predicted by TargetScan 7.1. (**B**) Luciferase activity of SPC-A1 cells co-transfected with either wild-type or mutant SOCS4 3′-UTR and miR-1290 mimic or miR-NC. SOCS4 expression was detected by qRT-PCR (**C**) and western blot (**D**) in SPC-A1 cells transfected with miR-1290 mimic or miR-NC. (**E**) Cell viability in SPC-A1 cells co-transfected with sh-SOCS4 or sh-NC and anti-miR-1290 or anti-miR-NC was detected by CCK-8 assay. (**F**) Invasiveness of SPC-A1 cells co-transfected with sh-SOCS4 or sh-NC and anti-miR-1290 or anti-miR-NC was detected by transwell invasion assay. Representative images are shown on the left, and invasive cell number based on 10 randomly selected fields was shown on the right. Data is presented as mean ± SD. ^*^P < 0.05, ^**^P < 0.01, ^****^P < 0.0001, ^#^*P <* 0.05 compared with sh-SOCS4 + anti-miR-1290 or sh-NC + anti-miR-NC group.

To explore the molecular mechanisms of the pro-tumor role of miR-1290 in LADC, potential targets of miR-1290 were analyzed with the TargetScan 7.1 predication database since that miRNA exerts effects mainly by suppressing translation of target genes. As shown in Figure 4A, SOCS4, a major member of the SOCS family, was identified as a candidate target of miR-1290 for further validation. To verify the target gene predicated, dual-luciferase reporter assay was performed in SPC-A1 cell line. The results showed that co-transfection of miR-1290 mimic significantly reduced the activity of luciferase reporter plasmid containing the wild-type, but not a mutant, 3′-UTR of SOCS4 (Figure 4B). Additionally, qRT-PCR and western blot analysis demonstrated that transfection of miR-1290 mimic in SPC-A1 cells significantly suppressed the expression of SOCS4 at both the mRNA and protein levels compared to miR-NC group (Figure 4C, 4D). Altogether, these data suggested that miR-1290 inhibited SOCS4 expression by directly targeting its 3′-UTR.

### miR-1290 enhances cell proliferation and invasion by targeting SOCS4

To confirm the involvement of SOCS4 in the pro-tumor effects of miR-1290, plasmid carrying shRNA targeting SOCS4 (sh-SOCS4) or control shRNA (sh-NC) was co-transfected with anti-miR-1290 or anti-miR-NC into SPC-A1 cells for further experiments. CCK-8 assay indicated that repression of SOCS4 promoted cell proliferation, while miR-1290 downregulation inhibited cell proliferation (Figure 4E). Moreover, the invasiveness of SPC-A1 cells transfected with sh-SOCS4 plasmid was higher than that of control, in contrast, miR-1290 downregulation weaken cell invasiveness (Figure 4F). Additionally, SOCS4 silencing reversed the negative effect of miR-1290 downregulation on cell proliferation and invasion (Figure 4E, 4F). These results indicated that miR-1290 enhanced LADC cell proliferation and invasion by targeting SOCS4.

### miR-1290 activates the JAK/STAT3 and PI3K/AKT signaling pathways by targeting SOCS4

**Figure 5:**
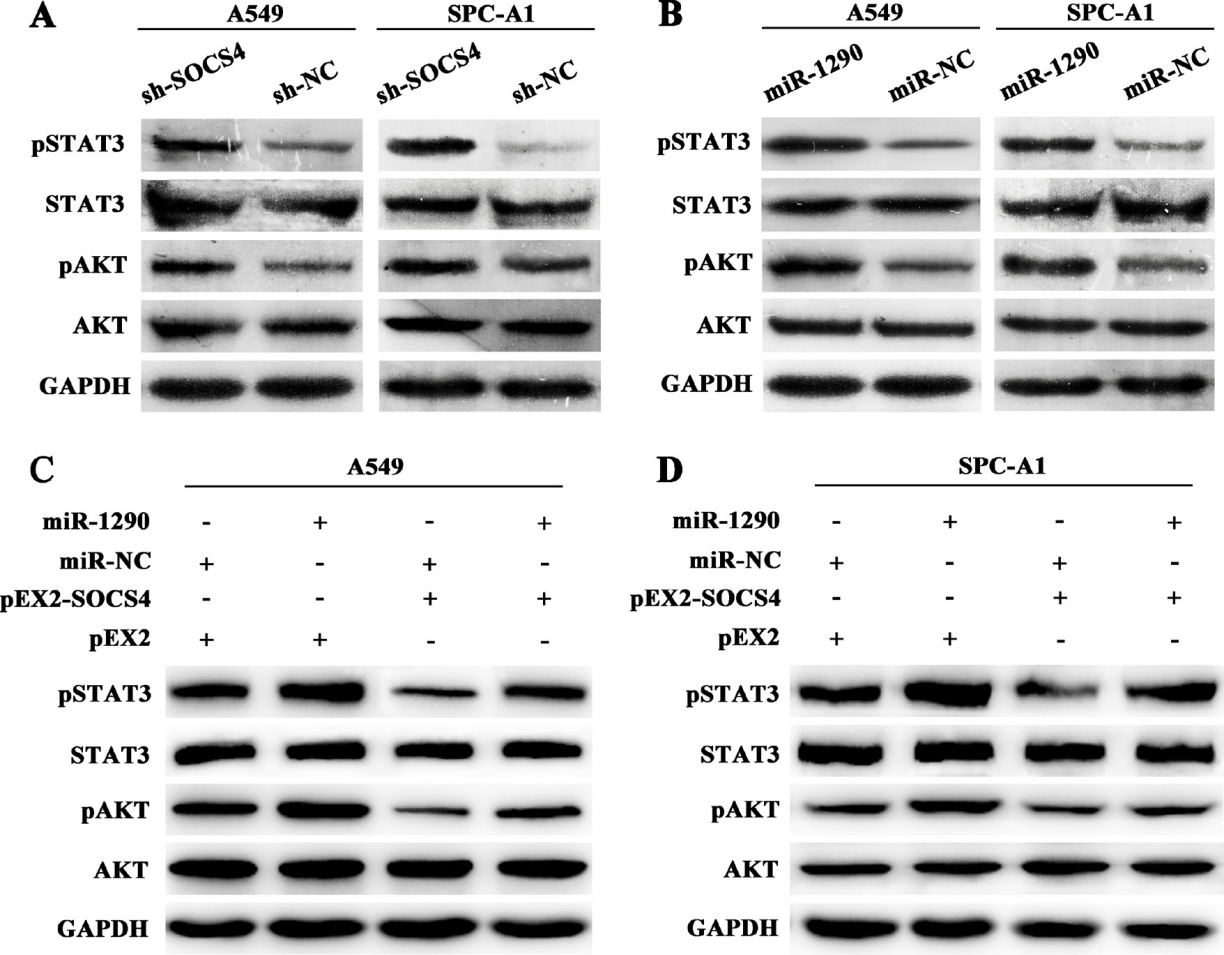
miR-1290 activates the JAK/STAT3 and PI3K/AKT signaling pathways by targeting SOCS4 (**A**) Phosphorylated (p)STAT3, STAT3, pAKT, and AKT expressions in A549 and SPC-A1 cells transfected with sh-SOCS4 or sh-NC plasmid were detected by western blot. (**B**) pSTAT3, STAT3, pAKT, and AKT expressions in A549 and SPC-A1 cells transfected with miR-1290 mimic or miR-NC were detected by western blot. (**C**) pSTAT3, STAT3, pAKT, and AKT expressions in A549 cells co-transfected with pEX2-SOCS4 or pEX2 and miR-1290 mimic or miR-NC was detected by western blot. (**D**) pSTAT3, STAT3, pAKT, and AKT expressions in SPC-A1 cells co-transfected with pEX2-SOCS4 or pEX2 and miR-1290 mimic or miR-NC was detected by western blot.

To clarify the possible mechanism underlying the pro-tumor effects of miR-1290, we further explored singaling pathway downstream of SOCS4. Western blot showed that SOCS4 downregulation resulted in increased levels of phosphorylated (p)STAT3 and pAKT but not of total STAT3 or AKT (Figure 5A). Consistently, transfection of miR-1290 mimic increased expression of pSTAT3 and pAKT but not of total STAT3 or AKT (Figure 5B). Furthermore, miR-1290 upregulation reversed the negative effect of SOCS4 overexpression on JAK/STAT3 and PI3K/AKT signaling pathways, indicated as increased expression of pSTAT3 and pAKT compared with control (Figure 5C, 5D). These results indicated that miR-1290 activated the JAK/STAT3 and PI3K/AKT signaling pathways by targeting SOCS4.

### SOCS4 is downregulated in lung adenocarcinoma tissues and negatively correlates with poor clinical outcomes

**Figure 6:**
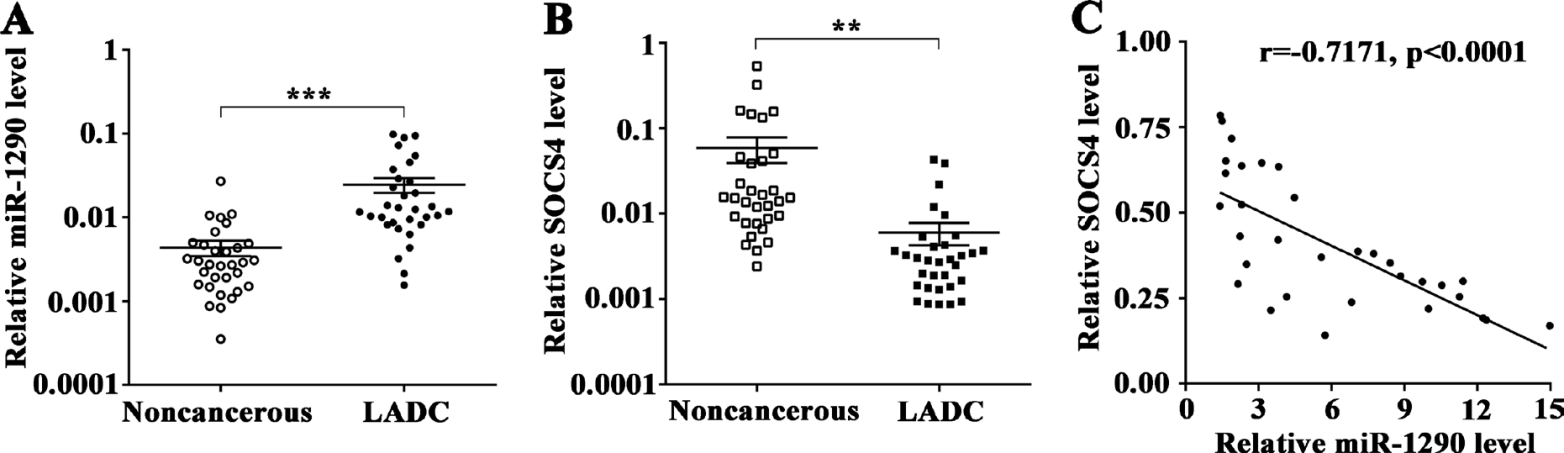
SOCS4 levels is inversely correlated with miR-1290 levels in lung adenocarcinoma tissues (**A**) miR-1290 levels in 32 pairs of LADC tissues and matched noncancerous lung tissues were detected by qRT-PCR. (**B**) SOCS4 levels in 32 pairs of LADC tissues and matched noncancerous lung tissues were detected by qRT-PCR. (**C**) Correlation between miR-1290 and SOCS4 levels in LADC tissues. Error bars represent SD. ^**^P < 0.01, ^***^P < 0.001.

To further explore whether miR-1290-mediated suppression of SOCS4 in LADC is clinically relevant, we assessed miR-1290 and SOCS4 levels in 32 pairs of LADC tissues and matched noncancerous lung tissues. qRT-PCR results showed that miR-1290 expression was significantly higher in LADC tissues than in noncancerous lung tissues (Figure 6A). In contrast, SOCS4 expression was downregulated in LADC tissues (Figure 6B). As shown in Figure 6C, SOCS4 levels were inversely correlated with miR-1290 levels in LADC tissues. To better understand the potential biological significance of SOCS4 in LADC, we evaluated the correlations between SOCS4 levels and clinicopathological characteristics in patients with LADC. We found that SOCS4 levels were negatively correlated with advanced clinical stage and lymphatic metastasis, and there was no significant correlation between SOCS4 levels and age, gender, smoking or tumor diameter (Table 1).

**Table 1:** Correlation between SOCS4 levels and clincopathological parameters in lung adenocarcinoma

Variables	Cases	SOCS4 level		*P* value
		Down-regulated	Up-regulated	
Age(years)				
≤ 60	18	10	8	1.000
> 60	14	8	6	
Gender				
Male	23	15	8	0.132
Female	9	3	6	
Smoking history				
Yes	17	10	7	1.000
No	15	8	7	
Tumor diameter(cm)				
≤3	16	9	7	1.000
>3	16	9	7	
Staging				
I + II	23	10	13	0.044
III + IV	9	8	1	
Lymphatic metastasis				
Yes	14	11	3	0.036
No	18	7	11	

## DISCUSSION

miRNA profiles have been established for multiple cancers including LADC [13]. Conspicuous changes of miRNA expression uncovered the essential role of these molecules in regulation of cancers. miR-1290 was first reported in human embryonic stem cells [16], and is upregulated in some cancers, including pancreatic cancer [17], esophageal squamous cell carcinoma [18] and colon cancer [19]. Li et al. identified miR-1290 as a potential marker for early detection of pancreatic cancer, and high serum miR-1290 level was correlated with poor prognosis. *In vitro* assays showed miR-1290 promoted cell proliferation and invasion [17]. Another publication by Zhang *et al.* indicated that miR-1290 was tumor-initiating, cell-specific RNA, which was a key driver of tumorigenesis and metastasis in NSCLC [20]. To date, there are few studies clarifying the role of miR-1290 in LADC and the molecular mechanism involving in it. No doubt that more recognition of miR-1290 in LADC is likely to shed light on cancer diagnosis and therapy.

As is well-known, sustaining cell proliferation is one of the basic traits of tumor. Here, we found that miR-1290 promoted cell proliferation. Moreover, subcutaneous xenograft model in nude mouse showed that larger tumors developed in miR-1290 overexpression group than in control, indicating that miR-1290 promoted tumor growth. Progression of cell cycle through mitosis is crucial in governing cell proliferation. Our results showed that miR-1290 increased the percentage of cells in G2/M phase and decreased the percentage in the G0/G1 peak, implying that miR-1290 facilitated cell cycle progression. Apoptosis is regarded as a natural obstacle to cancer development [21], nevertheless, apoptosis is frequently blunt in tumors at an advanced stage [21, 22]. Our datas showed that miR-1290 significantly dampened cell apoptosis. Activation of invasion and metastasis is a hallmark of cancer [23]. Here, Transwell assay showed that miR-1290 accelerated cell invasion *in vitro*, furthermore, bioluminescence imaging analysis showed that miR-1290 reinforced tumor invasion and metastasis in nude mice. These results revealed that miR-1290 functioned as a pro-tumor factor in LADC.

In a bid to explore the mechanisms underlying the effects of miR-1290 in LADC, we next got down to identify the target genes of miR-1290. Bioinformatics analysis hinted a potential target gene, SOCS4, a major member of the SOCS family. SOCS4 negatively regulates cell signal transduction, predominantly by controlling growth factor receptor signaling [24, 25]. SOCS4 was further verified as a target gene of miR-1290 by luciferase reporter assay. Ulteriorly, miR-1290 decreased SOCS4 expression at both the mRNA and protein levels. Moreover, an inverse correlation was observed between miR-1290 and SOCS4 expression in LADC tissues. Clinicopathological correlation analysis showed that SOCS4 expression was negatively correlated with higher clinical stage (*P =* 0.039) and lymph node metastasis (*P =* 0.025). Similar correlation was found for serum or tissue miR-1290 in NSCLC in our previous study [15]. Functional assays demonstrated that the inhibitory effects of miR-1290 downregulation on proliferation and invasion were diminished by reduced SOCS4. Indeed, SOCS4 was previously shown to be inversely associated with tumor node metastasis in breast cancer [26], and another research found that SOCS4 was attenuated in gastric cancer tissues and SOCS4 hypermethylation was related to poor prognosis [27]. Our datas also indicated inhibition of cell proliferation and invasion *in vitro* following miR-1290 downregulation. In keeping with our findings, Kim et al. demonstrated that inhibition of miR-1290 suppressed cell proliferation and invasion of NSCLC *in vitro* [28]. Zhang et al. showed that downregulation of miR-1290 inhibited tumor initiation and tumor growth of NSCLC [20]. These results implicated that miR-1290 may be an effective therapeutic target in LADC.

SOCS4 negatively regulates cell signaling via several mechanisms: degradation of receptors or proteins via the ubiquitin-proteasome pathway; competition with other signaling molecules for receptor phosphotyrosine residues; and inhibition of JAK tyrosine activity [29]. The JAK/STAT3 pathway plays an important role in lung cancer for its close link with proliferation, metastasis and prognosis [30–32]. The PI3K/AKT pathway is activated early in lung carcinogenesis and impacts on various biological behaviors such as proliferation, apoptosis, invasion and metastasis [33, 34]. A structural study showed that SOCS4 reduced STAT3 signaling by increasing EGFR degradation [25]. We hypothesized that SOCS4 might modulate the JAK/STAT3 and PI3K/AKT pathways, therefore serves as a tumor suppressor downstream of miR-1290 in LADC. We showed for the first time via functional assays that SOCS4 regulated the JAK/STAT3 and PI3K/AKT signaling pathways in LADC, as indicated by the fact that reducing SOCS4 level increased pSTAT3 and pAKT expression. According to a study in mice, a decrease in SOCS4 level intensified STAT3 activity preceding epithelial tumor initiation [35]. Consistently, here, miR-1290 upregulation resulted in increased pSTAT3 and pAKT levels. Moreover, miR-1290 upregulation reversed the negative effect of SOCS4 overexpression on JAK/STAT3 and PI3K/AKT signaling pathways. These results indicated that miR-1290 activated the JAK/STAT3 and PI3K/AKT pathways by targeting SOCS4. Jie et al. showed that miR-944 affected NSCLC proliferation and invasion by targeting SOCS4 [36], suggesting a crucial role of SOCS4 in NSCLC.

In conclusion, our results showed that miR-1290 activated the JAK/STAT3 and PI3K/AKT pathway by targeting SOCS4, and thereby promoted LADC cell proliferation, invasion and metastasis. The findings improve our understanding of LADC tumorigenesis and development, which may provide new insights to lung adenocarcinoma therapy.

## MATERIALS AND METHODS

### Human lung adenocarcinoma tissues

Thirty-two pairs of human lung adenocarcinoma tissues and noncancerous lung tissues were obtained from patients who underwent surgical resection at the First Affiliated Hospital, Nanjing Medical University (Nanjing, China). All specimens were immediately frozen in liquid nitrogen and stored at −196° C until analysis. Clinicopathological information for all of the patients was available. Lung adenocarcinoma was staged according to the seventh edition of the tumor-node-metastasis classification for lung cancer [37]. This study was approved by the medical ethics committee of the Institutional Review Board of the hospital. Standard written consent was obtained from each patient.

### Cell culture and transfection

Human bronchial epithelial cell line BEAS-2B and human lung adenocarcinoma cell line A549 were cultured in Dulbecco’s modified Eagle’s medium (DMEM; HyClone, USA) containing 10% fetal bovine serum (FBS; Gibco, USA) and 1% antibiotics at 37° C in a 5% CO_2_ incubator. Human lung adenocarcinoma cell line SPC-A1 were cultured in RPMI-1640 medium (HyClone) containing 10% FBS (Gibco) and 1% antibiotics at 37° C in a 5% CO_2_ incubator.

A549 and SPC-A1 cells were transfected with 100 nM miR-1290 mimic (miR-1290) or mimic control (miR-NC); 200 nM miR-1290 inhibitor (anti-miR-1290) or inhibitor control (anti-miR-NC) (Ribobio, Guangzhou, China); or 5 μg plasmid carrying shRNA targeting SOCS4 (sh-SOCS4) or control shRNA (sh-NC); or 6 μg pEX2-SOCS4 or pEX2 (GenePharma, Shanghai, China) using Lipofectamine 2000 (Invitrogen, Carlsbad, CA, USA ) according to the manufacturer’s protocol.

miR-1290 gene was inserted into a lentiviral vector containing the sequence encoding green fluorescence protein. Luciferase-labeled SPC-A1 cells were seeded in a 24-well plate (2.0 × 10^5^ cells/well), and incubated with the lentiviral vector containing miR-1290 gene for 24 h according to the manufacturer’s protocol. The cells were selected using medium containing 3.0 μg/ml puromycin (Sigma-Aldrich, St Louis, MO, USA) for one month, and individual clones that stably expressed the vector genes were isolated and cultured in medium containing 2.5 μg/ml puromycin for further experiments. Infection efficiency after lentiviral vector exposure was determined by fluorescence microscopy (Olympus, Tokyo, Japan) and qRT-PCR.

### Cell proliferation assay

Cells (2.0 × 10^4^ cells/well) were seeded in 96-well plates and treated with 100 nM miR-1290 mimic or miR-NC. Cells were subjected to Cell Counting Kit-8 assay (Beyotime, Guangzhou, China) according to the manufacturer’s instructions at 48 h after transfection. Optical density values at 450 nm were detected after incubation for 0.5–4 h. Each value represents the mean of five repeated measurements. Cell viability was determined across three independent experiments. Each assay was repeated for three times.

### Flow cytometry analysis of cell cycle and apoptosis

For cell cycle analysis, cells were harvested, washed twice with cold phosphate buffered saline (PBS) and fixed in 75% ethanol overnight at 4° C. The cells were then washed twice with cold PBS. Next, the cells were stained with propidium iodide solution (propidium iodide, 3.4 mM Tris-Cl, 100 μg/ml RNase A, 0.1% Triton X-100) for 20 minutes at room temperature in the dark. DNA content was then measured with a FACSCalibur flow cytometer (BD Biosciences, New York, USA), and the percentage of cells in G0/G1, S, and G2/M phase were analyzed using ModFit software (BD Biosciences). Each assay was repeated for three times.

Cell apoptosis was analyzed using Annexin V/PE and 7-AAD apoptosis detection kit (BD Biosciences). Briefly, cells were harvested after transfection, washed twice with cold PBS and resuspended in Annexin-binding buffer at a density of 10^6^ cells/ml. The cells were then stained with Annexin V/PE and 7-AAD for 15 minutes at room temperature in the dark. Cell apoptosis was detected using a FACSCalibur flow cytometer (BD Biosciences) and analyzed by Flowjo software (BD Biosciences). Each assay was repeated for three times.

### *In vitro* cell invasion assays

Transwell invasion assays were performed in 24-well plates containing 8.0-μm pore size chamber inserts (Corning, New York, USA) coated with MaxGel extracellular matrix mixture (Sigma-Aldrich) according to the manufacturer’s instructions. Cells (2.0 × 10^5^/well) were placed onto the upper insert pre-coated with MaxGel extracellular matrix mixture and cultured in serum-free medium. The lower chamber contained 700 μl medium supplemented with 10% FBS. After incubation for 24 h, any cells that had not migrated were removed carefully, and cells on the lower surface of the membrane were fixed with 95% ethanol and stained with 1% crystal violet. Cells adhering to the membrane were imaged and counted under at least 10 random fields using an inverted microscopy (Olympus). Each assay was repeated for three times.

### miRNA target prediction

Putative miRNA target genes were predicted using TargetScan 7.1 (www.targetscan.org).

### RNA isolation and qRT–PCR assay

Total RNA was isolated with a miRcute miRNA isolation kit (Tiangen, Beijing, China). Levels of hsa-miR-1290 (5′-UGGAUUUUUGGAUCAGGGA-3′) were determined using TaqMan miRNA assays (Applied Biosystems, Foster City, USA) with specific RT primers and probes. Reverse-transcribed complementary DNA was synthesized with 10 ng total RNA using a TaqMan MicroRNA reverse transcription kit (Applied Biosystems). U6 was used as an internal control. Quantitative real-time PCR for SOCS4 was performed with SYBR Premix DimerEraser (TaKaRa, Japan). Approximately 500 ng of total RNA was reverse-transcribed using PrimeScript RT Master Mix Perfect Real Time (TaKaRa). mRNA expression was normalized to β-actin mRNA expression.

The primer sequences were as follows:

SOCS4 forward: 5′-GGCAGTGTTTTCCAATAAAAG-3′; SOCS4 reverse: 5′-GGTGGG AAAGGACACTTAT-3′; β-actin forward: 5′-TGGCCCCAGCAATGAA-3′;β-actin reverse: 5′-CTAAGTCATAGTCCGCCTAGAAGCA-3′.qRT-PCR was performed using a 7500 PCR amplification system (Applied Biosystems). The qRT-PCR reactions were performed in triplicate. Expression levels were calculated using the 2^−ΔΔCT^ method. Each assay was repeated for three times.

### Western blot

Cell lysates were prepared using ice-cold immunoprecipitation lysis buffer (Beyotime) and protein concentrations were determined with a bicinchoninic acid protein assay kit (Beyotime). Proteins (50 μg) were loaded into and separated using 10% SDS-PAGE gels, and transferred to PVDF membranes (Millipore, Billerica, USA). After being blocked with 5% skimmed milk for 1 h, the membranes were incubated with the antibodies (anti-SOCS4 [R&D Systems, USA], anti-pSTAT3 [Cell Signaling Technology, MA, USA], anti-STAT3 [Cell Signaling Technology], anti-pAKT [Cell Signaling Technology], or anti-AKT [Cell Signaling Technology]) overnight at 4° C as per the manufacturer’s instructions. After being washed three times with Tris-buffered saline containing 0.1% Tween 20, the membranes were incubated with horseradish peroxidase (HRP) conjugated secondary antibody for 1 h, and protein bands were visualized using chemiluminescent HRP Substrate (Millipore). GAPDH (Beyotime) was used as an internal control. Each assay was repeated for three times.

### Luciferase reporter assay

SPC-A1 cells were seeded in 24-well plates and transfected with Lipofectamine 2000 reagent (Invitrogen, USA) 24 h later. miR-1290 mimic or miR-NC (100 nM) were co-transfected with 200 ng luciferase reporter plasmid (GENEray, Shanghai, China). Luciferase activity in lysates was measured after 48 h using a Dual-Luciferase Reporter Assay kit (Promega, WI, USA) according to the manufacturer’s instructions, and was normalized to renilla luminescence. Each assay was repeated for three times.

### Animal studies

All animal studies were permitted by the National Institutional Animal Care and Use Committee and approved by the regional ethics committee.

For the *in vivo* tumorigenesis study, 1 × 10^7^ SPC-A1 cells expressing miR-1290 or miR-NC were subcutaneously injected into the right flank of 5-week-old BALB/c-nu/nu nude mice (*n =* 3). Tumor size was monitored every week and measured using a caliper. Tumor volume (V) was calculated by the formula V (mm^3^) = (L × W^2^) /2, in which L represents the longest dimension and W represents the shortest dimension of the tumor. After five weeks, the mice were sacrificed and the tumor xenografts were fixed in 10% formalin and stored at −196° C immediately.

For the *in vivo* invasion and metastasis study, 5 × 10^6^ cells were injected into the tail vein of BALB/c-nu/nu nude mice (*n =* 3). The distribution and intensity of SPC-A1 cells were monitored by bioluminescence imaging with an *In Vivo* Imaging system (IVIS Spectrum, PerkinElmer, USA) at week 0, 1, 3, 5 and 7. Ten minutes before conducting live imaging, each mouse was injected with 150 μg/g D-luciferin (Fanbo Biochemicals, China) into the peritoneal cavity and then anesthetized with isoflurane. Luminescence values for regions of interest were analyzed using Living Imaging software.

### Statistical analysis

Data are presented as mean ± standard deviation unless otherwise stated. Quantitative data were analyzed using the Student’s *t*-test (two-tailed) to compare two groups or one-way analysis of variance (ANOVA) to compare more than two groups. The relationship between miR-1290 and SOCS4 expression was evaluated by Spearman’s correlation. The significance of relationships between SOCS4 expression and clinicopathological factors was determined by Fisher’s exact test. *P <* 0.05 was considered statistically significant. Analysis was performed with SPSS 19.0.

## Abbreviations

lADC: lung adenocarcinoma
SOCS4: suppressors of cytokine singaling 4
